# Supplementary material for: Identification of CAND1 as a DNA-dependent protein kinase-regulated coactivator of androgen receptor and the ARv7 splice variant
Source: PLoS One. 2026 May 14;21(5):e0349130. doi: 10.1371/journal.pone.0349130 (PMC13175348; doi:10.1371/journal.pone.0349130)
Supplement: S1 File — This file contains S1-S4 Tables, Legends for S1-S5 Fig, and S1-S5 Fig. (DOCX) [file pone.0349130.s001.docx]

**S1 File. Supplementary Material.** This file contains S1-S4 Tables, Legends for S1-S5 Figures, and S1-S5 Figures.

**Supplementary Tables**

**S1 Table. Antibodies used for immunoblotting (IB), immunodepletion (ID) or ChIP.**

| **Antibody:** | **Source:** | **Dilution used:** | **Vendor:** | **Catalog number:** | **Application:** | **Research Resource Identifier (RRID):** | **Reference for prior use**  **(PMID number):** |
| --- | --- | --- | --- | --- | --- | --- | --- |
| AR (441)  Used to detect both AR and ARv7 on same blot | mouse monoclonal | 1:5000 | Santa Cruz Biotechnology | sc-7305 | IB | AB_626671 | 35354884 |
| AR (N-20) | rabbit polyclonal | 1:5000 | Santa Cruz Biotechnology | sc-816 | IB | AB_1563391 | 35354884 |
| p300 | rabbit polyclonal | 1:1000 | Santa Cruz Biotechnology | sc-585 | IB | AB_2231120 | 23850489 |
| CBP | rabbit polyclonal | 1:250 | Santa Cruz Biotechnology | sc-369 | IB | AB_631006 | 23850489 |
| SRC-3 (custom made) | mouse monoclonal | 1:1000 | Courtesy of Dr. Ping Yi | N.A. | IB | AB_2868421 | 29748621 |
| CARM1 | rabbit polyclonal | 1:1000 | Bethyl Laboratories | A300-421A | IB | AB_2068452 | 33412112 |
| NELF-E | rabbit polyclonal | 1:1000 | Bethyl Laboratories | A301-914A | IB | AB_1524077 | 27282391 |
| ANP32A | goat polyclonal | 1:1000 | Santa Cruz Biotechnology | sc-5652 | IB | AB_2289588 | 21317927 |
| CAND1 | rabbit polyclonal | 1:1000 | Bethyl Laboratories | A302-901A | IB | AB_10663486 | 29499133 |
| CNOT2 | rabbit polyclonal | 1:1000 | Bethyl Laboratories | A302-562A | IB | AB_2034831 | 35660762 |
| HDAC1 | rabbit polyclonal | 1:1000 | Bethyl Laboratories | A300-713A | IB | AB_533395 | 29775582 |
| DNA-PKcs | goat polyclonal | 1:5000 | Santa Cruz Biotechnology | sc-1552 | IB | AB_2172847 | 23850489 |
| DNA-PKcs (pS2056) | rabbit polyclonal | 1:5000 | Abcam | ab18192 | IB | AB_869495 | 20406977 |
| Ku70 | rabbit polyclonal | 1:1000 | Bethyl Laboratories | A302-624A | IB | AB_10554672 | 35581289 |
| SPT6 | rabbit polyclonal | 1:1000 | Bethyl Laboratories | A300-801A | IB | AB_577215 | 33542242 |
| NELF-B | rabbit polyclonal | 1:1000 | Bethyl Laboratories | A301-912A | IB | AB_1524092 | 28868519 |
| SUG1 | rabbit polyclonal | 1:1000 | Bethyl Laboratories | A300-791A | IB | AB_577239 | 29748621 |
| E6-AP | mouse monoclonal | 1:500 | Santa Cruz Biotechnology | sc-166688 | IB | AB_2211810 | This study |
| DNA-PKcs | mouse monoclonal |  | Thermo Scientific | MA5-13404 | ID | AB_11004133 | 23850489 |
| Anti-mouse IgG |  |  | Santa Cruz Biotechnology | sc-2025 | ID | AB_737182 |  |
| AR | rabbit polyclonal |  | Active Motif | 39781 | ChIP | AB_2793341 | 35354884 |
| CAND1 | rabbit polyclonal |  | Bethyl Laboratories | A302-901A | ChIP | AB_10663486 | This study |
| Anti-rabbit IgG |  |  | Bethyl Laboratories | P120-101 | ChIP | AB_479829 |  |

**S2 Table. List of Primers for PCR, ChIP-qPCR, and Cell-free Transcription.**

| **Primer:** | **Sequence (Listed 5’-3’):** | **Use:** | **Reference for prior use (PMID number):** |
| --- | --- | --- | --- |
| E4BioF | biotin-gatgaccctgctgattggtt | Generating 3xARE-E4 fragment | 23850489 |
| E4BioR | biotin-aaccgtattaccgcctttga |  |  |
| E4F | ccgtgtcgagtggtgtttt | Measuring E4 mRNA from cell-free transcription assays | 23850489 |
| E4R | aacaacatacagcgcttcca |  |  |
| *PSA* enhancer For | gacctactctggaggaacatattg | ChIP for AR binding site upstream of *PSA* gene | 35354884 |
| *PSA* enhancer Rev | ggcttgcttactgtcctagataa |  |  |
| *FKBP5* For | aggtcaactcttgctggaac | ChIP for AR binding site upstream of *FKBP5* gene | 35354884 |
| *FKBP5* Rev | actgggagttgtttggtctg |  |  |

**S3 Table. List of commercially available and custom designed siRNAs.**

(A) Commercially available siRNAs:

| **Target siRNA:** | **Vendor:** | **Catalog number:** | **Reference for prior use (PMID number):** |
| --- | --- | --- | --- |
| siDNA-PKcs pool | Santa Cruz Biotechnology | sc-35200 | 23850489 |
| siRNA-A pool (Control for siDNA-PKcs) | Santa Cruz Biotechnology | sc-37007 | 23850489 |
| Mission siRNA Universal negative control #1 (siCtrl for ANP32A ,CAND1, SRC-3, and MED1) | Sigma | SIC001-10NMOL | 29775582 |

(B) Custom designed siRNAs (all were from Sigma):

For each gene, all three siRNAs were pooled 1:1:1 as then used as a pool.

| **Target siRNA:** | **Sequence (Listed 5’-3’):** | **Reference for prior use (PMID number):** |
| --- | --- | --- |
| siANP32A – 1 | CACCUCAAUCGCAAACUUA | This study |
| siANP32A – 2 | CCUAUUGUGAUUUGACUGU | This study |
| siANP32A – 3 | GAUGAUGACUAAGUGGAAU | This study |
| siCAND1 – 1 | CUUUGAAACUGGGUACUCU | This study |
| siCAND1 – 2 | CCAAGUAGAGACAAUUGUA | This study |
| siCAND1 - 3 | CUUACAAGUGCAAUAGCAA | This study |
| siSRC-3-1 | CAGACAUGAUCUUUCAGGA | 29775582 |
| siSRC-3-2 | CAAACCAGCAGAAUAUCAU | 29775582 |
| siSRC-3-3 | GCUAUUCUUGGUGAUCUGA | 29775582 |
| siMED1-1 | CUCAAAUGGCUUCUUCUAA | This study |
| siMED1-2 | CAGCAUUGGCCCAGAUGUA | This study |
| siMED1-3 | CAUGUCAGGAGGAGAGUUU | This study |

**S4 Table. List of primers for RT-qPCR gene expression analysis.**

| **Primers:** | **Sequence (Listed 5’-3’):** | **Roche Universal Probe Library Probe:** |
| --- | --- | --- |
| GAPDH Forward | agccacatcgctcagacac | #60 |
| GAPDH Reverse | gcccaatacgaccaaatcc |  |
| PSA Forward | cctgtccgtgacgtggat | #75 |
| PSA Reverse | cagggttgggaatgcttct |  |
| FKBP5 Forward | acaatgaagaaagccccaca | #55 |
| FKBP5 Reverse | caccattccccactcttttg |  |
| TMPRSS2 Forward | tcacaccagccatgatctgt | #4 |
| TMPRSS2 Reverse | actgtcaccctggcaagaat |  |
| ETS2 Forward | cagcgtcacctactgctctg | #27 |
| ETS2 Reverse | agtcgtggtctttgggagtc |  |
| EDN2 Forward | tcctggctcgacaaggagt | #29 |
| EDN2 Reverse | ccgtaaggagctgtctgttca |  |
| ANP32A Forward | tgtataaaagaacatctgcaccagt | #51 |
| ANP32A Reverse | aactcaaagtttggctcttctga |  |
| CAND1 Forward | tcgagacatttcaagtattggtctt | #65 |
| CAND1 Reverse | gcagctaatgcagagccact |  |
| DNA-PKcs Forward | acacgttcattggcgagag | #1 |
| DNA-PKcs Reverse | tgaagactgggcttcagtacc |  |
| EXTL2 Forward | aaaaattaaacttcaacacaatgagg | #14 |
| EXTL2 Reverse | ccaccaaagataatcgaagca |  |
| RASSF3 Forward | gacctccttcttcaggagagc | #37 |
| RASSF3 Reverse | ggtttccttctctttctcaacatc |  |
| ARv7 Forward | cttgtcgtcttcggaaatgtt | #14 |
| ARv7 Reverse | tcagggtctggtcattttga |  |

**Supplementary Figure Legends**

**S1 Fig. Control assays testing recombinant AR and ARv7 DNA binding efficiencies, titrating ARv7 and AR in ARE DNA pulldown assay using HeLa nuclear extract (NE) for optimal coregulator recruitment, endogenous AR-ARE DNA pulldowns using LNCaP NE, and validation of additional ARv7 candidates from HeLa NE identified by mass spectrometry.** A) Immunoblots of AR and ARv7 after incubating recombinant protein and DNA together for 10 min to demonstrate similar DNA-binding efficiencies when equivalent amounts of protein are added. 2 µg of AR and 0.25 µg ARv7 provided optimal coregulator recruitment in the ARE pulldown assay using nuclear extracts (see below). * represents the amount of AR or ARv7 bound to DNA after conducting the standard/optimal ARE pulldown with HeLa NE, followed by ice-cold 2x NETN and 1x D-PBS washes. B) *Left*, Immunoblots showing ARE pulldowns using HeLa NE with increasing amounts of recombinant AR and ARv7 to demonstrate optimal recruitment of a known AR coactivator, CARM1. Significantly less ARv7 (0.25 µg) is required for CARM1 recruitment compared to AR (2 µg). ARv7 amounts (left to right) are as follows: 0.125 µg, 0.25 µg, 0.5 µg, 0.75 µg, 1 µg, while AR amounts are as follows: 0.125 µg, 0.25 µg, 0.5 µg, 1 µg, and 2 µg. HDAC1 is used as a loading control. *Right*, Increasing amount of ARv7 lead to reduced coregulator recruitment as shown for CARM1 and the ubiquitin E3 ligase, E6-AP. ARv7 amounts used here are as follows: 0.25 µg and 1 µg. C) ARE DNA pulldown from LNCaP nuclear extract (-/+) R1881 to validate the recruitment of several identified mass spectrometry candidates to endogenously expressed AR in this prostate cancer cell line. D) Immunoblotting validation of additional ARv7 candidate coregulators (SPT6, NELF-B, and SUG1) from HeLa NE identified from MS data in Fig 1C.

**S2 Fig. Analysis of *ARv7*, *ANP32A*, and *CAND1* mRNA levels and ARv7 target gene *EDN2* expression upon doxycycline (dox) treatment or siRNA-mediated knockdown in LNCaP doxycycline (dox)-inducible ARv7 cells.** A) *ARv7* mRNA and an ARv7 target gene (*EDN2*) are induced by 200 ng/ml dox treatment in LNCaP dox-inducible ARv7 cells. mRNA was analyzed by RT-qPCR and normalized to *GAPDH*. B) *ANP32A* and *CAND1* knockdown efficiencies in LNCaP dox-inducible ARv7 cells. mRNA was analyzed by RT-qPCR and normalized to *GAPDH*. C) *ARv7* mRNA expression levels were not reduced by ANP32A and CAND1 knockdown in LNCaP dox-inducible ARv7 cells. N.S., not significant.

**S3 Fig. Dual knockdown of ANP32A and CAND1 significantly reduces AR and ARv7 target gene expression in LNCaP dox-inducible ARv7 cells.** A) Dual knockdown of ANP32A and CAND1 significantly reduced R1881 (AR)-driven target gene expression of *PSA* and *TMPRSS2*. P-values: for *PSA,* p = 0.0020, for *TMPRSS2*, p = 0.0030. B) Dual knockdown of ANP32A and CAND1 significantly reduced dox (ARv7)-driven target gene expression of *PSA* and *TMPRSS2*. P-values: for *PSA*, p = 0.037, for *TMPRSS2*, p = 0.050. In both panels, mRNA was analyzed by RT-qPCR and normalized to *GAPDH*. *p < 0.05, **p < 0.005.

**S4 Fig. Control experiments for the inhibition of DNA-PKcs in cell-free transcription or DNA-PKcs knockdown efficiency in LNCaP dox-inducible ARv7 cells.** A) Cell-free transcription showing no effects of the DNA-PK inhibitor NU7441 on basal transcription reactions (i.e., without any recombinant AR or ARv7 present). N.S., not significant. E4 mRNA was measured by RT-qPCR. B) RT-qPCR analyses showing DNA-PK siRNA knockdown efficiency in LNCaP dox-inducible ARv7 cells. mRNA levels were normalized to *GAPDH*.

**S5 Fig. Additional tested AR target genes are inhibited and *ARv7* mRNA is not reduced upon DNA-PKcs knockdown.** A) Knockdown of DNA-PKcs significantly reduced the expression of AR selective target genes *EXTL2* and *RASSF3*, supporting DNA-PK function as an AR coactivator. P-values: for *EXTL2*, p = 0.050, for *RASSF3*, p = 0.0091. B) Knockdown of DNA-PKcs did not reduce *ARv7* mRNA levels suggesting that reduction in ARv7 target gene expression was not due to less receptor being present. P-value for ARv7 increase upon DNA-PK knockdown: p = 0.048. In both panels, mRNA levels were analyzed by RT-qPCR and normalized to *GAPDH*. *p < 0.05, **p < 0.01.

**S1 Fig**


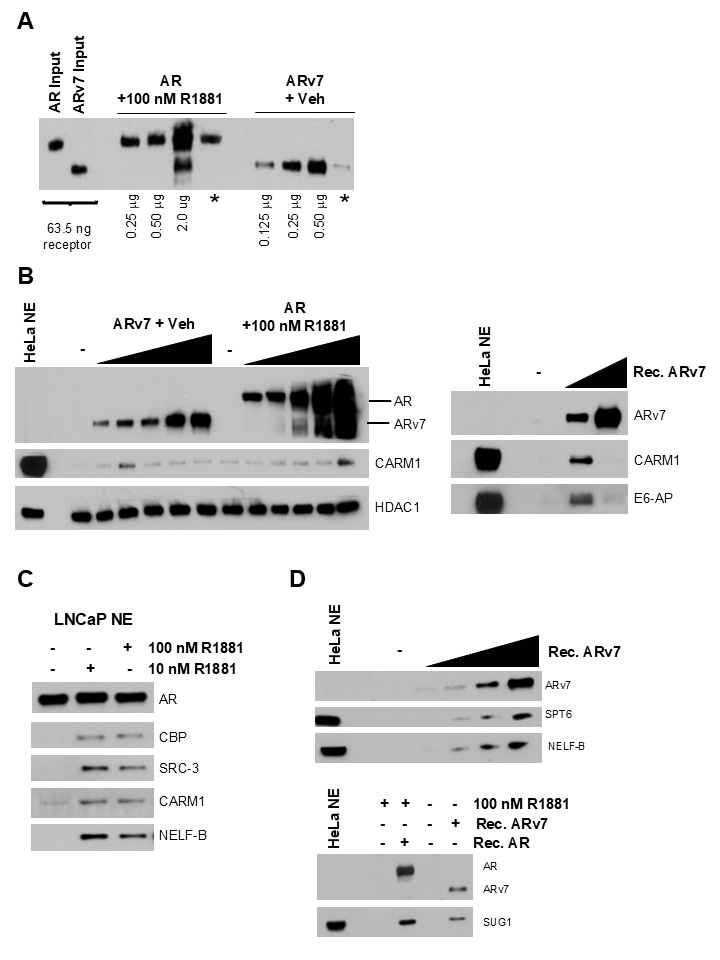


**S2 Fig**

**
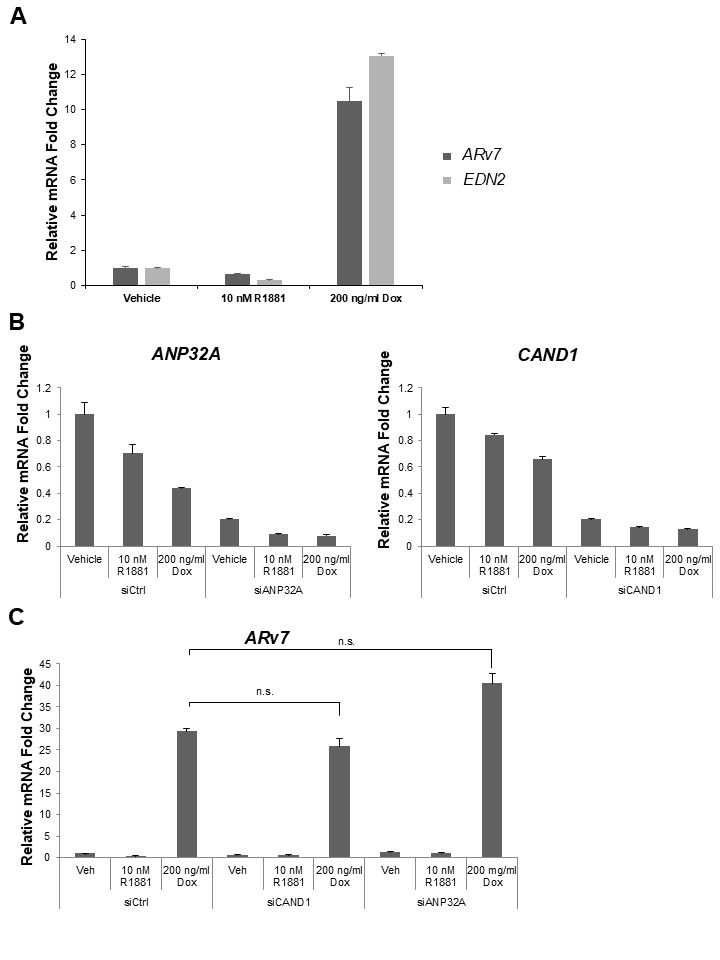
**

**S3 Fig**

**
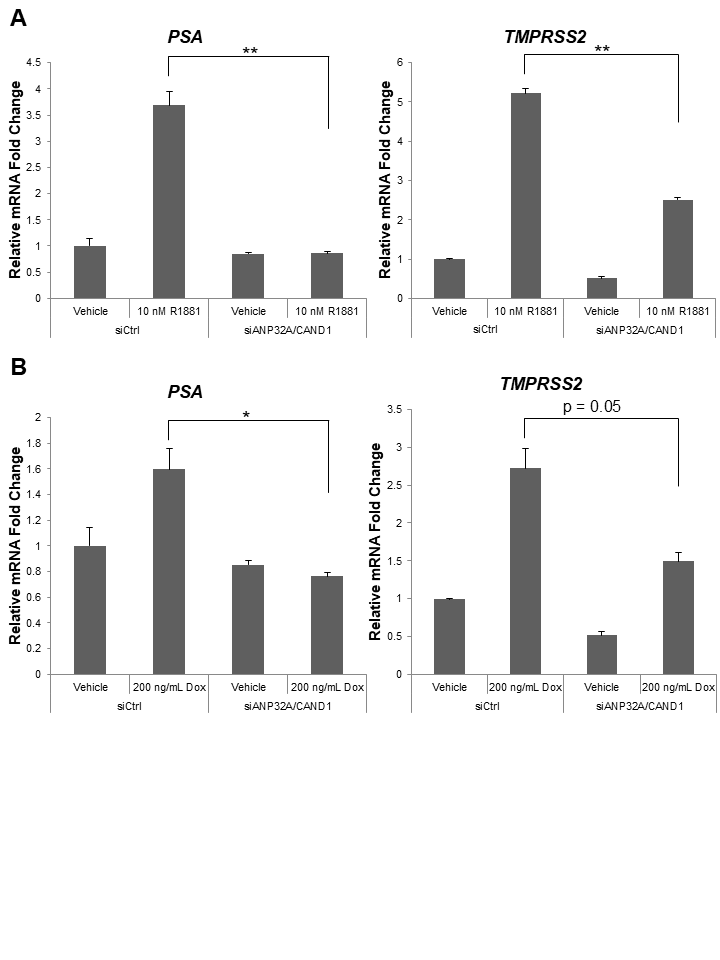
**

**S4 Fig**

**
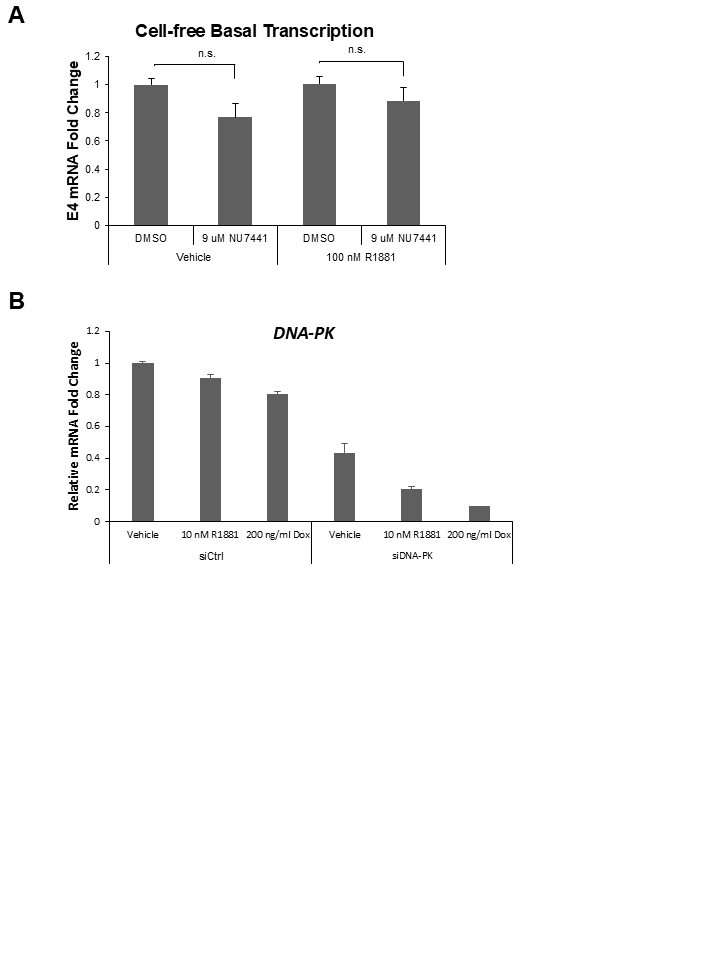
**

**S5 Fig**

**
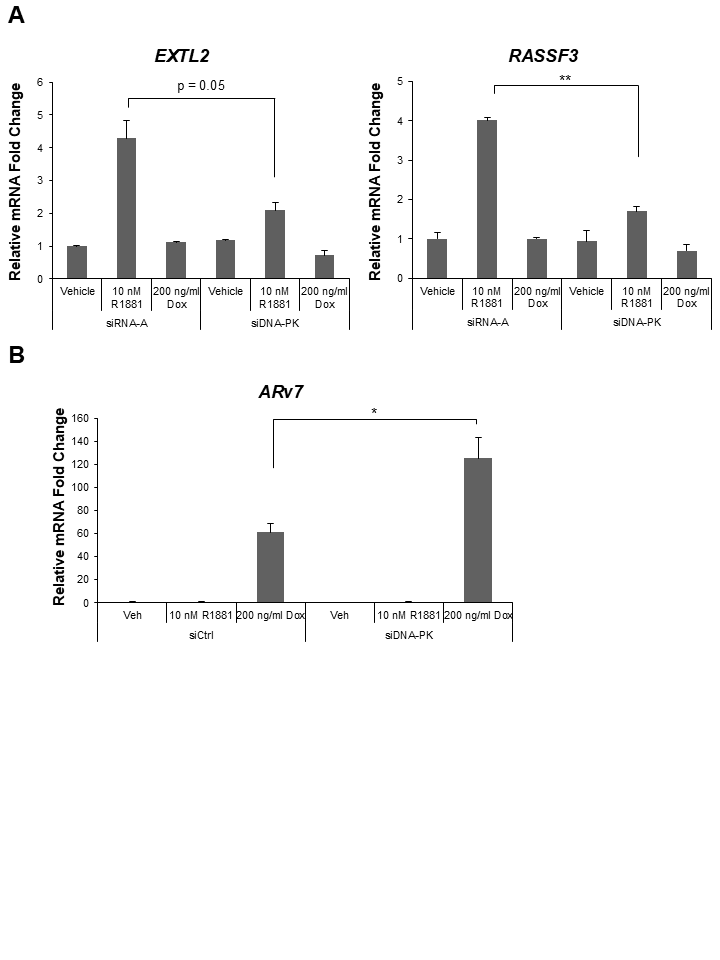
**
