# Supplementary material for: Identification of CAND1 as a DNA-dependent protein kinase-regulated coactivator of androgen receptor and the ARv7 splice variant
Source: PLoS One. 2026 May 14;21(5):e0349130. doi: 10.1371/journal.pone.0349130 (PMC13175348; doi:10.1371/journal.pone.0349130)
Supplement: S6 Fig — (PDF) [file pone.0349130.s003.pdf]

Fig 1D: -/+ AR

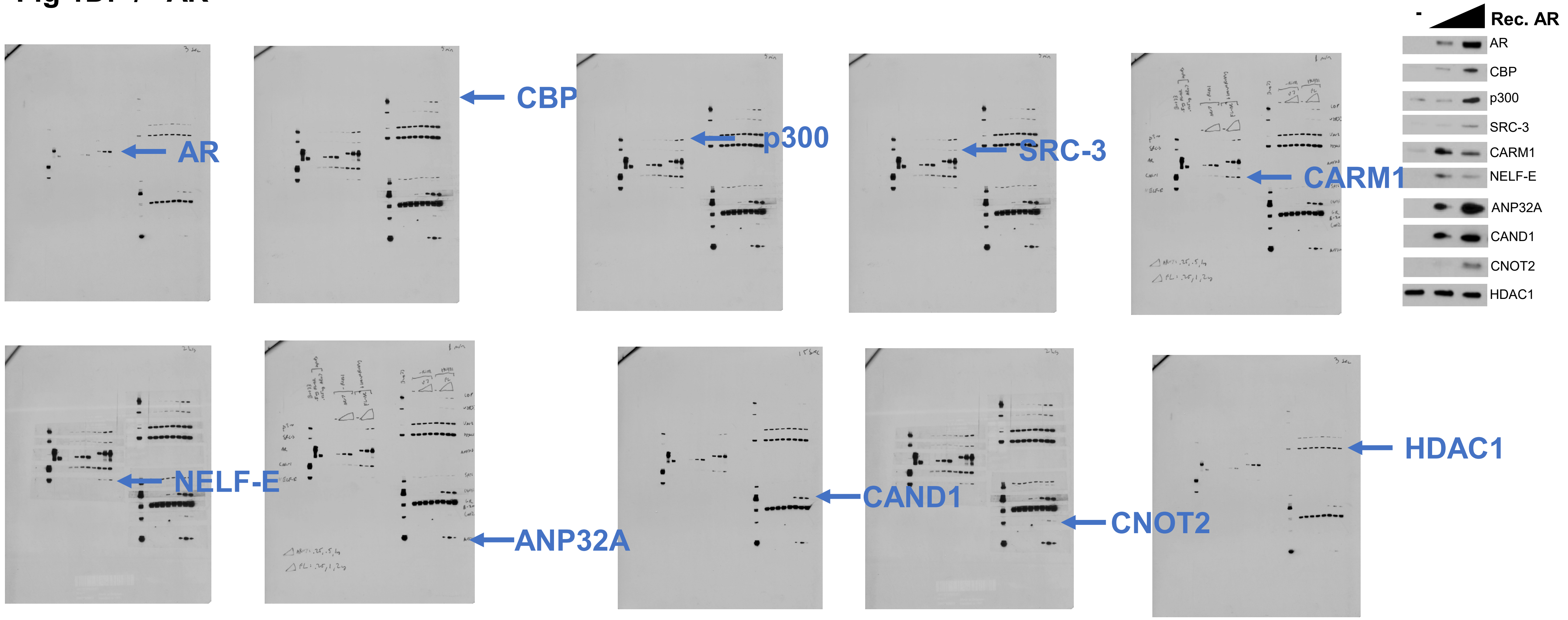
AR
CBP
p300
SRC-3
CARM1
NELF-E
ANP32A
CAND1
CNOT2
HDAC1
-
Rec. AR
AR
CBP
p300
SRC-3
CARM1
NELF-E
ANP32A
CAND1
CNOT2
HDAC1

Fig 1D: -/+ ARv7

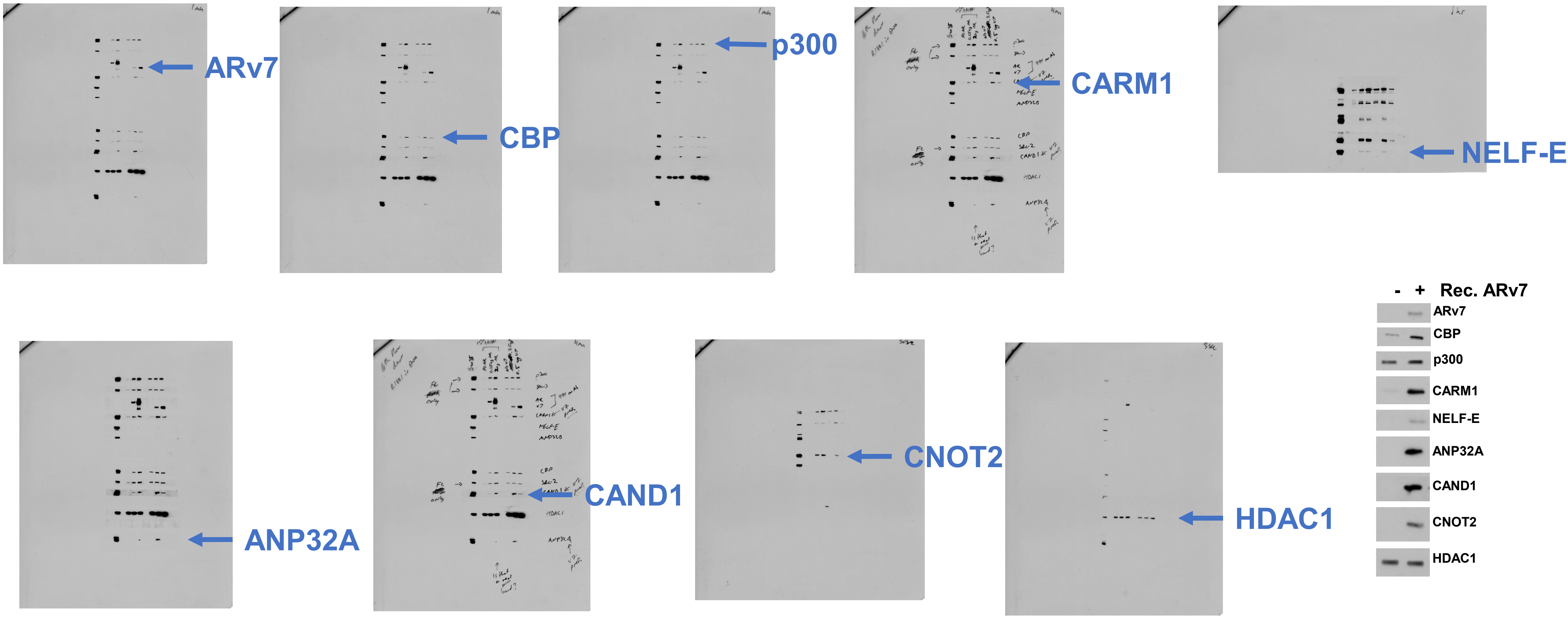
ARv7
CBP
p300
CARM1
NELF-E
ANP32A
CAND1
CNOT2
HDAC1
- + Rec. ARv7
ARv7
CBP
p300
CARM1
NELF-E
ANP32A
CAND1
CNOT2
HDAC1

Fig 1E

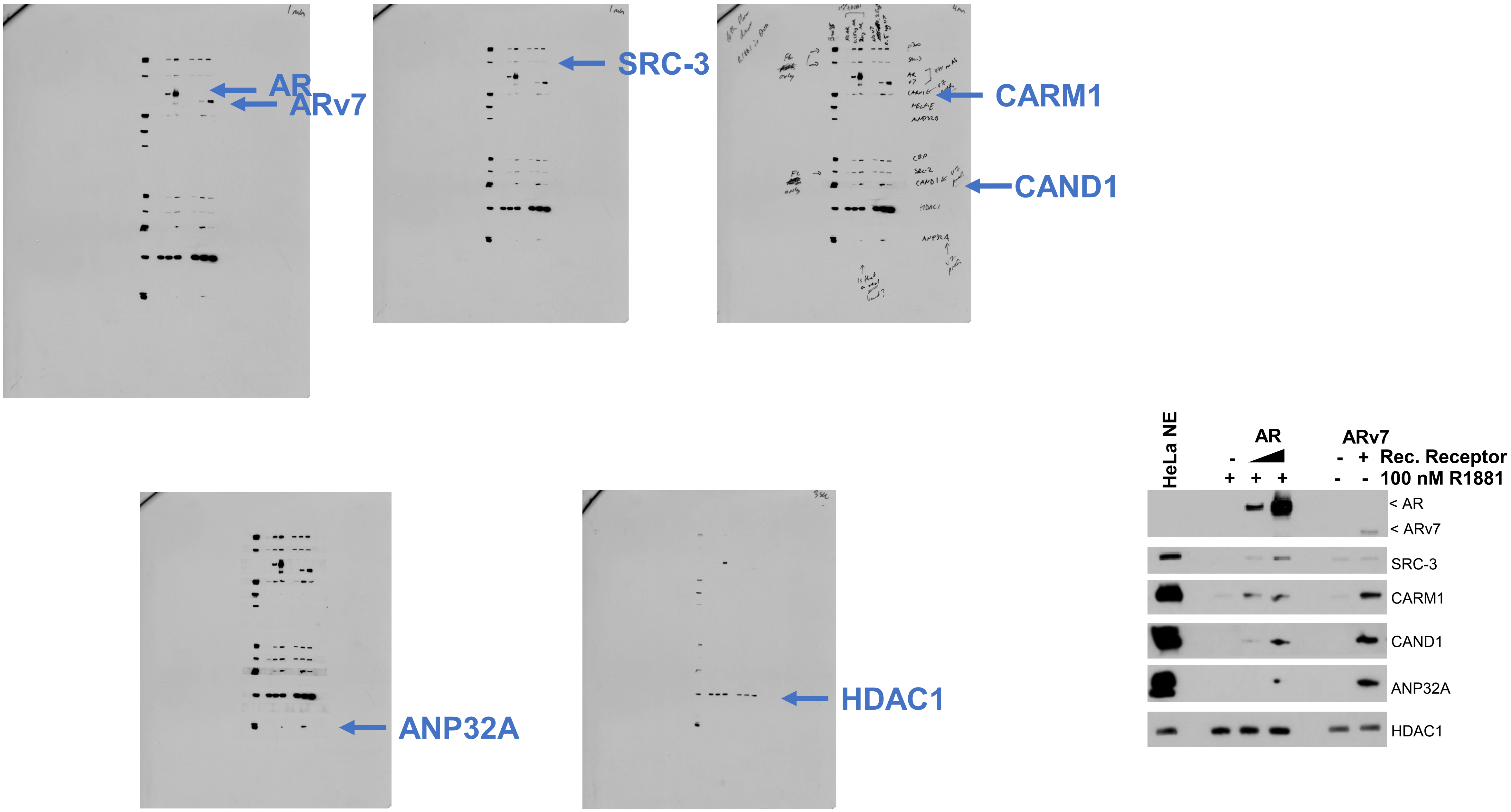
AR
ARv7
SRC-3
CARM1
CAND1
ANP32A
HDAC1
HeLa NE
AR
ARv7
- + Rec. Receptor
+ + + - - 100 nM R1881
< AR
< ARv7
SRC-3
CARM1
CAND1
ANP32A
HDAC1

Fig 5A

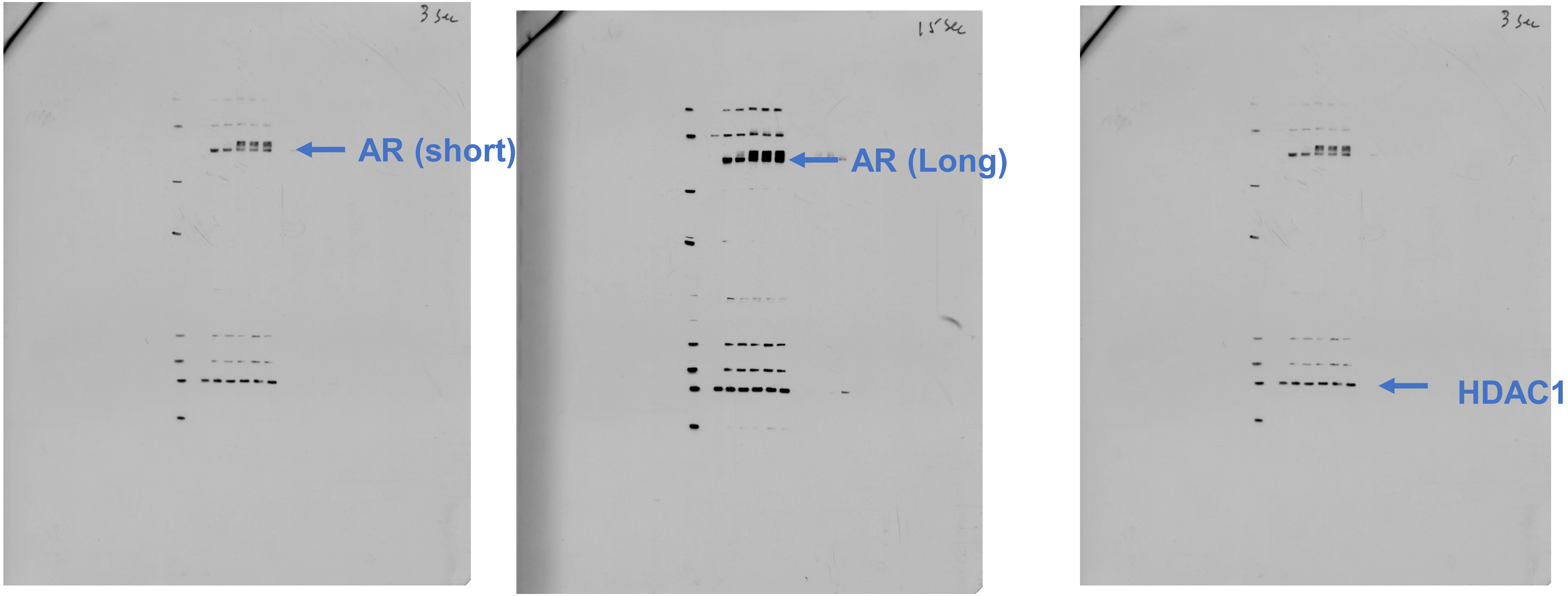
3 sec
AR (short)
15 sec
AR (Long)
3 sec
HDAC1

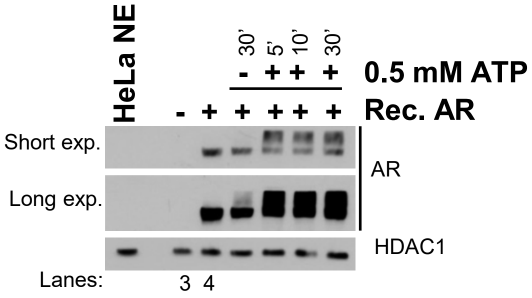
HeLa NE
30'
5'
10'
30'
- + + +
0.5 mM ATP
- + + + + + +
Rec. AR
Short exp.
Long exp.
AR
HDAC1
Lanes:
3 4

Fig 5B

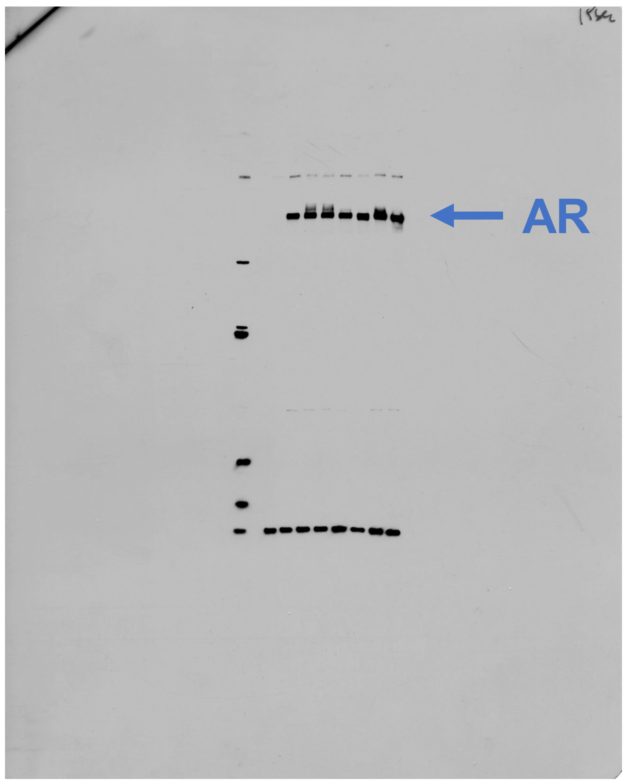

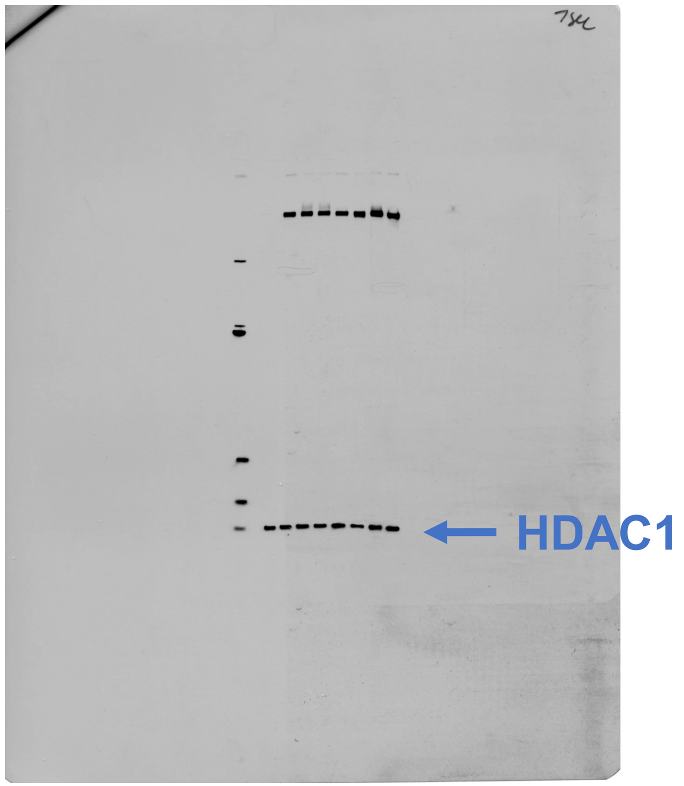

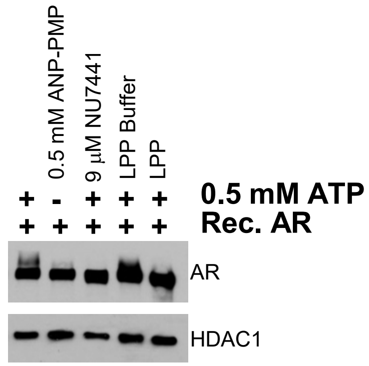

Lanes 3-7

Fig 5C

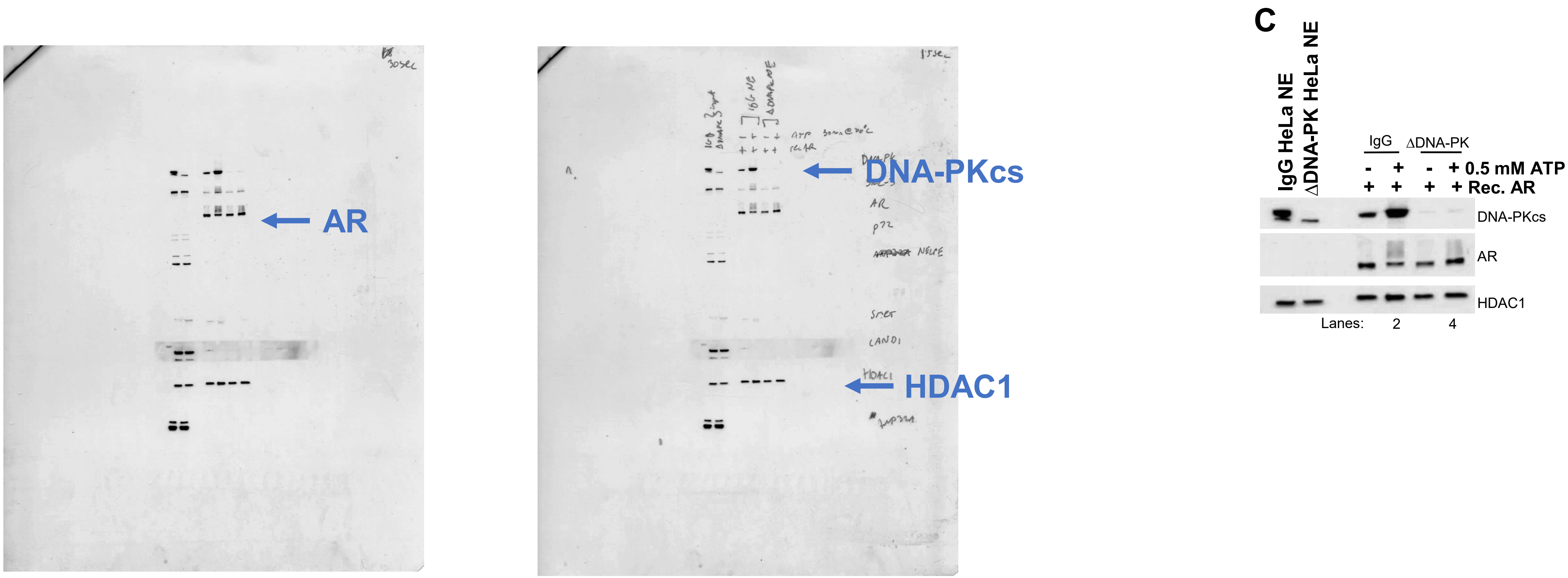
AR
DNA-PKcs
HDAC1
C
IgG HeLa NE
ΔDNA-PK HeLa NE
IgG
ΔDNA-PK
- + - + 0.5 mM ATP
+ + + + Rec. AR
DNA-PKcs
AR
HDAC1
Lanes: 2 4

Fig 5D

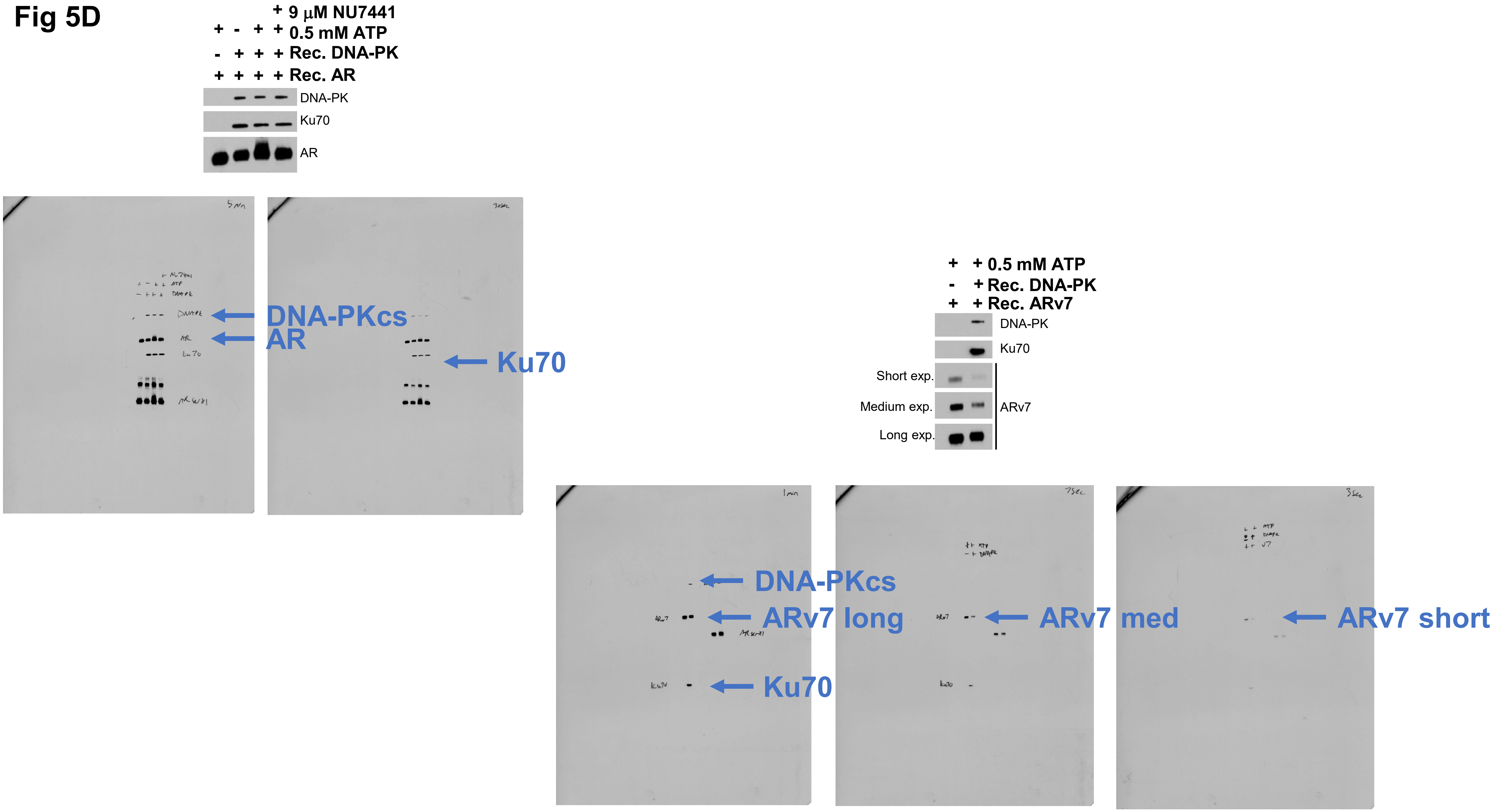
+ 9 µM NU7441
+ - + + 0.5 mM ATP
- + + + Rec. DNA-PK
+ + + + Rec. AR
DNA-PK
Ku70
AR
DNA-PKcs
AR
Ku70
+ + 0.5 mM ATP
- + Rec. DNA-PK
+ + Rec. ARv7
DNA-PK
Ku70
Short exp.
Medium exp.
Long exp.
ARv7
DNA-PKcs
ARv7 long
Ku70
ARv7 med
ARv7 short

Fig 5E

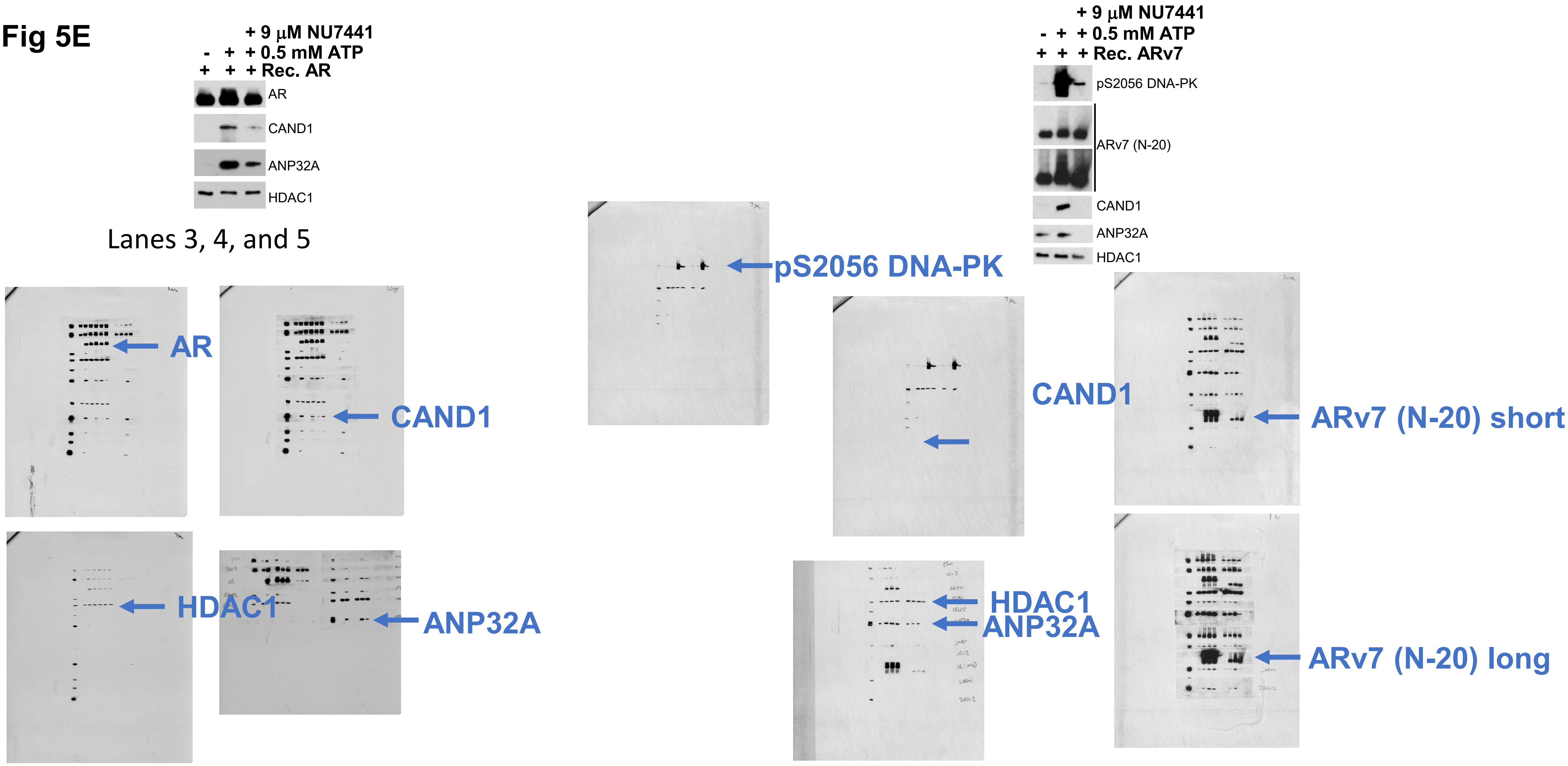

+ 9 μM NU7441
- + + 0.5 mM ATP
+ + + Rec. AR
AR
CAND1
ANP32A
HDAC1
Lanes 3, 4, and 5
AR
CAND1
HDAC1
ANP32A
pS2056 DNA-PK
CAND1
+ 9 μM NU7441
- + + 0.5 mM ATP
+ + + Rec. ARv7
pS2056 DNA-PK
ARv7 (N-20)
CAND1
ANP32A
HDAC1
ARv7 (N-20) short
HDAC1
ANP32A
ARv7 (N-20) long

S1A Fig

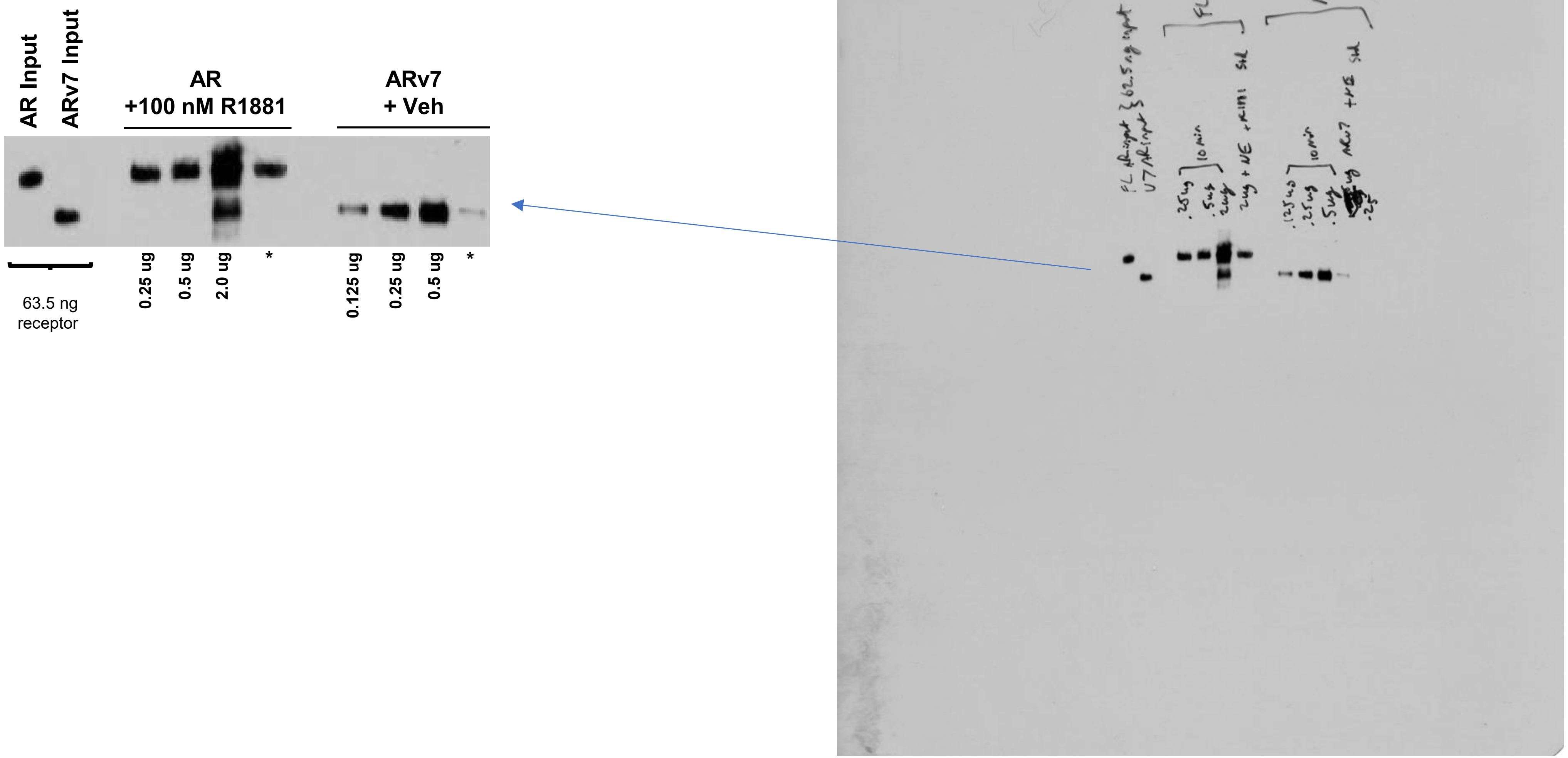
AR Input
ARv7 Input
AR
+100 nM R1881
ARv7
+ Veh
0.25 ug
0.5 ug
2.0 ug
*
0.125 ug
0.25 ug
0.5 ug
*
63.5 ng
receptor

S1B Fig

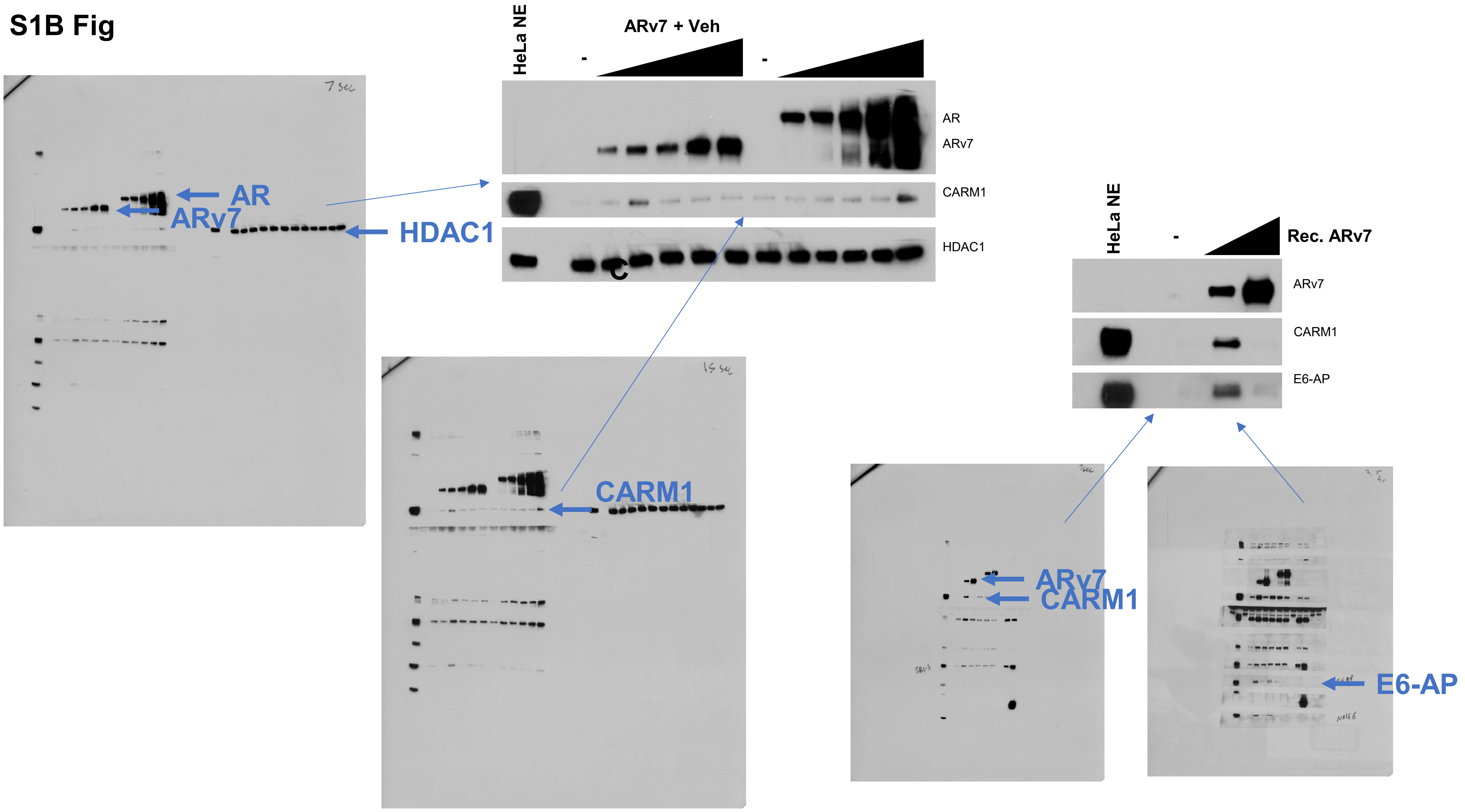
HeLa NE
ARv7 + Veh
-
-
AR
ARv7
CARM1
HDAC1
HeLa NE
-
Rec. ARv7
ARv7
CARM1
E6-AP
7 sec
AR
ARv7
HDAC1
15 sec
CARM1
ARv7
CARM1
E6-AP

S1C Fig

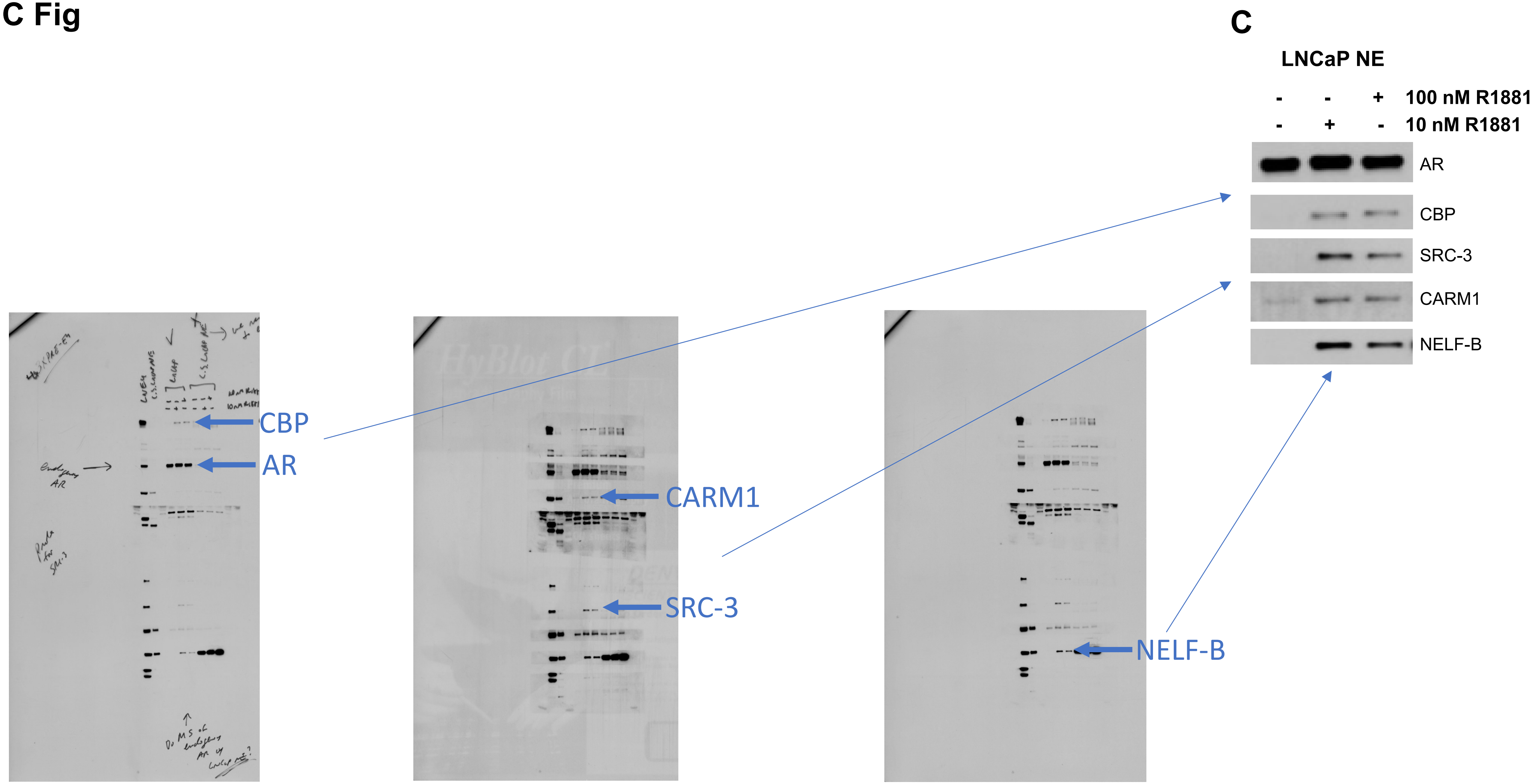
C
LNCaP NE
- - + 100 nM R1881
- + - 10 nM R1881
AR
CBP
SRC-3
CARM1
NELF-B
CBP
AR
CARM1
SRC-3
NELF-B

S1D Fig

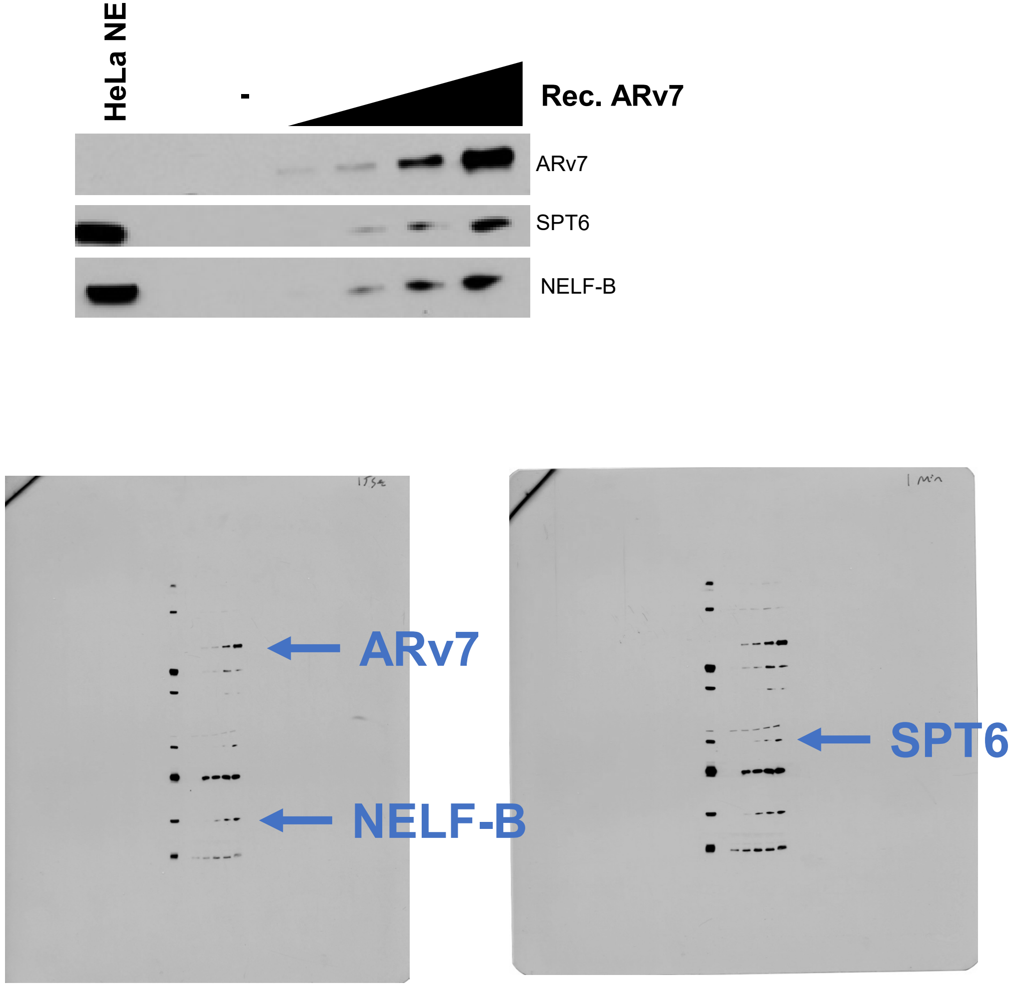

HeLa NE
-
Rec. ARv7
ARv7
SPT6
NELF-B
ARv7
NELF-B
SPT6

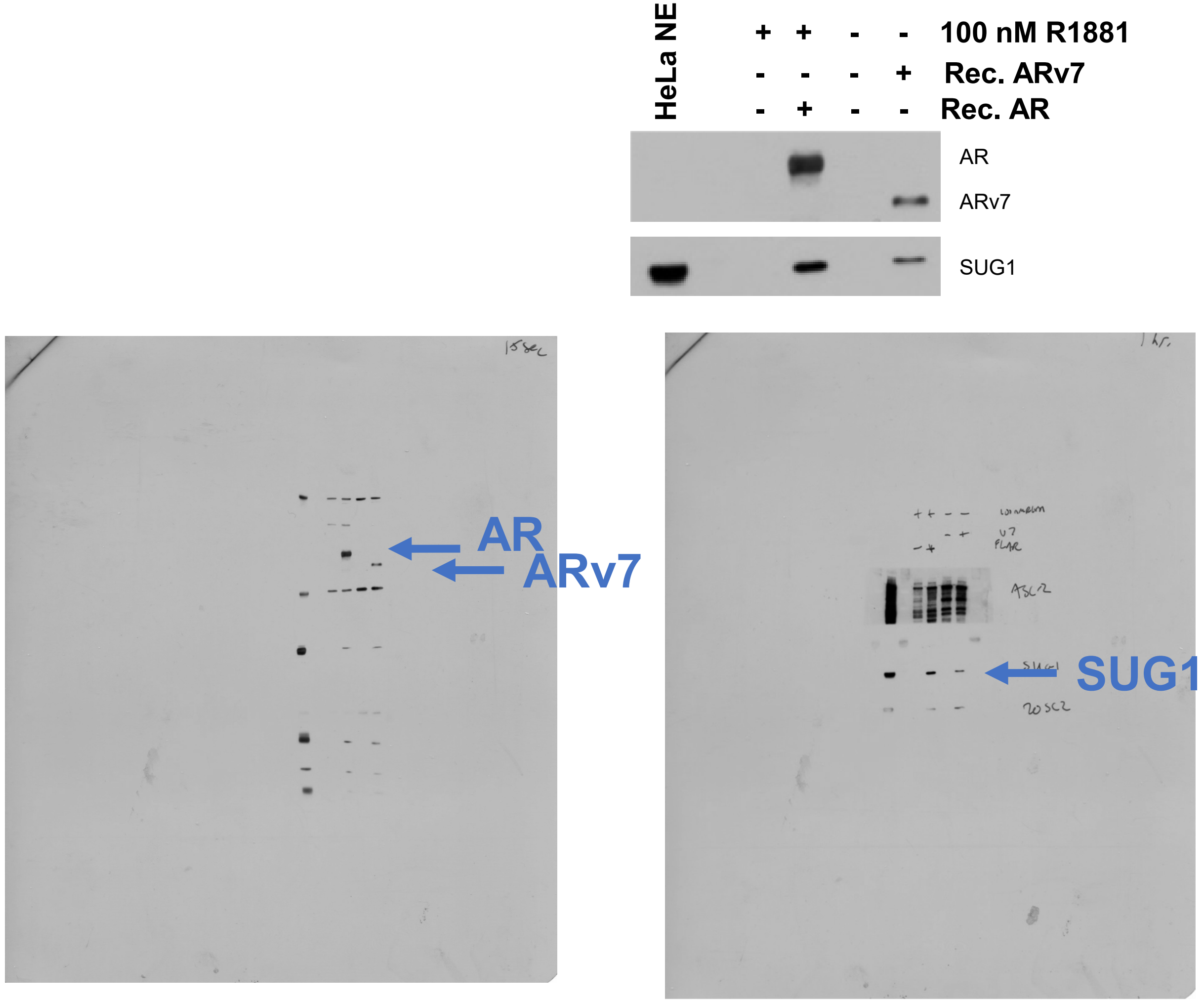

HeLa NE
+ + - - 100 nM R1881
- - - + Rec. ARv7
- + - - Rec. AR
AR
ARv7
SUG1
AR
ARv7
SUG1
